# Necroptosis-related lncRNA-based novel signature to predict the prognosis and immune landscape in soft tissue sarcomas

**DOI:** 10.1007/s00432-024-05682-w

**Published:** 2024-04-18

**Authors:** Qiuzhong Long, Zhengtian Li, Wenkang Yang, Ke Huang, Gang Du

**Affiliations:** 1 Department of Bone and Joint Surgery, The First Affiliated Hospital of Guangxi Medical University, Nanning, 530021 Guangxi China; 2 Guangxi Medical University, Nanning, Guangxi China; 3 Wuming Affiliated Hospital of Guangxi Medical University, Nanning, 530021 Guangxi China

**Keywords:** Soft tissue sarcomas, Necroptosis, Long noncoding RNAs, Immune landscape, Prognostic signature

## Abstract

**Background:**

Necroptosis-related long noncoding RNAs (lncRNAs) play crucial roles in cancer initiation and progression. Nevertheless, the role and mechanism of necroptosis-related lncRNAs in soft tissue sarcomas (STS) is so far unknown and needs to be explored further.

**Methods:**

Clinical and genomic data were obtained from the UCSC Xena database. All STS patients’ subclusters were performed by unsupervised consensus clustering method based on the prognosis-specific lncRNAs, and then assessed their survival advantage and immune infiltrates. In addition, we explored the pathways and biological processes in subclusters through gene set enrichment analysis. At last, we established the necroptosis-related lncRNA-based risk signature (NRLncSig) using the least absolute shrinkage and selection operator (LASSO) method, and explored the prediction performance and immune microenvironment of this signature in STS.

**Results:**

A total of 911 normal soft tissue samples and 259 STS patients were included in current study. 39 prognosis-specific necroptosis-related lncRNAs were selected. Cluster 2 had a worse survival than the cluster 1 and characterized by different immune landscape in STS. A worse outcome in the high-risk group was observed by survival analysis and indicated an immunosuppressive microenvironment. The ROC curve analyses illustrated that the NRLncSig performing competitively in prediction of prognosis for STS patients. In addition, the nomogram presents excellent performance in predicting prognosis, which may be more beneficial towards STS patients’ treatment.

**Conclusions:**

Our result indicated that the NRLncSig could be a good independent predictor of prognosis, and significantly connected with immune microenvironment, thereby providing new insights into the roles of necroptosis-related lncRNAs in STS.

**Supplementary Information:**

The online version contains supplementary material available at 10.1007/s00432-024-05682-w.

## Introduction

Soft tissue sarcomas (STS) are rare, heterogeneous malignant tumor arising from tissues of mesenchymal origin characterized by more than 70 distinct subtypes, including the muscle, adipose, and nerve sheath, and comprise <1% of adult cancer (Bourcier et al. 2019). Patients with advanced STS have a 5-year survival rate of approximately 27% over the last several decades, despite significant improvements in surgical procedures, radiotherapy, and chemotherapy (Bessen et al. 2019; Kim et al. 2019). In addition, those patients with primary cancer eventually affect up to 50% of patients with metastatic disease without early diagnosis, and this signifies a major obstacle to individualize treatment of STS patients (Ratan and Patel 2016). Therefore, there is an urgent need to develop more accurate diagnosis biomarkers and treatment strategies, including the new therapeutic targets. Given that most primary tumors have an innate apoptosis-resistant phenotype, the necroptosis, one of the programmed cell deaths, has become a promising strategy for the treatment of various solid cancers (Tang et al. 2020). Unlike other programmed apoptosis, necroptosis is a form of regulated cell death that is characterized by formation of the necrosome complex based on swelling injury (Seo et al. 2020). Necroptosis was reported to be related the regulation of T cell proliferation (Osborn et al. 2010; Bell et al. 2008) and macrophage survival (Hitomi et al. 2008), and might be involved in the remodeling of immune system. Some research had suggested that necroptosis in tumor microenvironment (TME) may promote tumor progression and metastasis (Wu and Zhou 2009; Liu et al. 2015; Seifert et al. 2016). Necroptosis could generate an immunosuppressive TME to promote malignancies at the same time, which prompted us to assume that necroptosis might be a potential immunotherapy target in STS (Gong et al. 2019).

On the other hand, long noncoding RNAs (lncRNAs) are defined as RNA transcripts longer than 200 nucleotides but without protein coding potential (St Laurent et al. 2015). LncRNAs have been reported to play key roles in molecular and cellular functions, and its dysregulation impacts on various cancer and metabolic diseases (Bhan et al. 2017; Ma et al. 2018; Robinson et al. 2020). Emerging evidence is demonstrating the important role of lncRNAs in cancer development, metastasis, and drug resistance by modulating diverse molecules (Ma et al. 2019; Peng et al. 2020; Xin et al. 2018). Despite this relevance to cancer, only a handful of lncRNAs had been reported as key regulators of necroptosis in other cancer, such as gastric cancer (Zhao et al. 2021), whereas still little is known about biological roles of necroptosis-related lncRNAs in STS. Therefore, exploration of necroptosis-related lncRNAs may aid the development of diagnostic tools and therapeutic strategy for STS.

Herein, we constructed a necroptosis-related lncRNA-based risk signatures (NRLncSig) to predict the prognosis of STS patients, and its specific prognostic value was comprehensively investigated by a range of bioinformatic methods. Furthermore, we also explored their role on the tumor-immune microenvironment (TME) in STS, thereby revealing the potential of NRLncSig as a novel prognostic biomarker and effective therapeutic target in STS patients.

## Materials and methods

### Patients and datasets

We obtained RNA sequencing (RNA-seq) data and corresponding clinical data of STS from the UCSC Xena database (https://xenabrowser.net/), which included the data of TCGA and GTEx database. The data of 263 STS tumor samples and 911 normal samples acquired from the UCSC Xena database were further processed into log 2(FPKM + 1). We included 259 STS patients with corresponding clinical data, of which 104 were leiomyosarcoma (LMS), 58 were dedifferentiated liposarcomas (DDLPS), 51 were undifferentiated pleomorphic sarcoma (UPS), 25 were myxofibrosarcomas (MFS), 10 were synovial sarcomas (SS), and 11 were other STS types. The clinicopathological characteristics of all the participants are presented in Table 1. We followed the methods of Zhang et al. (Zhang et al. 2021).

**Table 1:** Clinical characteristics of STS patients in the study

Characteristics (*n*; %)	TGCA-STS (*n* = 259)
Age	
≤60	128 (49.4)
>60	131 (50.6)
Gender	
Male	118 (45.6)
Female	141 (54.4)
Histological type	
DDLPS	58 (22.4)
LMS	104 (40.2)
MFS	25 (9.6)
SS	10 (3.9)
UPS	51 (19.7)
Other	11 (4.2)
Metastasis	
Yes	56 (21.6)
No	120 (46.3)
Unknown	83 (32.0)
Margin status	
Positive	73 (28.2)
Negative	136 (52.4)
Unknown	40 (15.4)
Recurrence	
Yes	29 (11.2)
No	143 (55.2)
Unknown	87 (33.6)
Radiotherapy	
Yes	74 (28.6)
No	179 (69.1)
Unknown	6 (2.3)

*DDLPS* dedifferentiated liposarcoma, *LMS* leiomyosarcoma, *MFS* myxofibrosarcoma, *SS* synovial sarcoma, *UPS* undifferentiated pleomorphic sarcoma

### Identification of necroptosis-related lncRNAs

Identifications of NRGs was made based on the current literature as well GSEA set, generating 67 selected NRGs (Table S1). All the mRNA expression data of NRGs were obtained from data of STS samples in TCGA cohort and GTEx cohort. To identify the necroptosis-related lncRNAs, we performed the Pearson correlation analysis. We defined these lncRNAs with correlation coefficients > 0.4 and *p* < 0.001 as necroptosis-related lncRNAs.

### Consensus clustering

With the help of “ConsensuClusterPlus” R package, we applied unsupervised clustering method towards STS patients and classified them into several clusters, and then repeated this procedure 1000 time to provide robust and accurate clustering. The optimal number of clusters was selected according to cophenetic, dispersion, and silhouette coefficients. Taking optimal number parameter, we classified samples into several clusters and keep low similarity and weak correlation among clusters as much as possible.

### Gene set enrichment analysis

GSEA was performed using GSEA software (version 4.0.3; http://software.broadinstitute.org/gsea/) to explore the function of all necroptosis-related lncRNA in different subclusters and risk groups. Gene sets with the *p* < 0.05 were considered significantly enriched.

### Construction and validation of risk signature

The 259 STS patients are randomized in a 1:1 ratio into train set establishing risk score and test set which to perform internal validation. Via the R package “glmnet”, we performed LASSO method which can minimize the risk of overfitting to select the final hub genes to construct the risk signature. Parameter λ is the tuning parameter chosen by tenfold cross-validation based on the minimum criteria. Integrating expression level of hub genes and corresponding regression coefficients, the risk score was calculated as follows: risk score = ∑ExplncRNAi*βi, where ExplncRNA represents the relative expression value of the hub necroptosis-related lncRNA, and *β* represents the regression coefficient. After randomly divided patients into the train set, test set, we separated cases in train set into high- and low-risk groups according to the median risk score. We performed both univariate and multivariate Cox analyses to this signature to explore its prognosis significance. In addition, integrating the risk score with other clinical characteristics including age and metastatic, we established a Nomogram as a prognosis tool to predict 1-, 3-, and 5-year survival of STS patients. The accuracy of this nomogram was evaluated using calibration curve (with 1000 bootstrap resamples).

### Immune microenvironment assessment

With the help of ESTIMATE algorithm, we evaluate the ratio of immune-stromal components in TME and outputs stroma, immune and ESTIMATE scores of each STS patient using the “estimate” R package. We carried out an immune cell infiltration analysis toward 22 immune cell subtypes by CIBERSORT algorithm. Applying CIBERSORT, a full-fledged immune cell estimating analysis algorithm, we assessed the relative quantity of immune cells in tissue samples based on the gene expression profiles. Correlation of the risk score and immune cell infiltration was analyzed by Spearman correlation analysis method.

### Investigation of the significance of the risk signature in predicting immune response and chemosensitivity

We conducted single-sample GSEA (ssGSEA) to quantify the activity of several important immune-related pathways via the “gsva” package (Mootha et al. 2003; Subramanian et al. 2005). After computing number of somatic mutations for each patient via Perl scripts, we generated TMB score and divided samples into high and low-TMB groups based on the median TMB value. We accordingly calculated half-inhibitory concentration (IC50) values of chemotherapeutic drugs for the further study of curative effect prediction. The investigation was conducted and the results were visualized through “pRRophetic” and “ggplot2” R packages.

### Statistical analysis

All statistical analyses and data processing were performed using R software (version 4.1.2). The difference analysis of the overall survival of between grouped patients were performed via Kaplan–Meier analyses and a Kaplan–Meier curve was generated. We applied time-dependent ROC curve analysis through “survivalROC” R package to assess ability to predict prognosis. Subgroups were analyzed to evaluate the stability of the risk signature in different groups. The differences between subgroups were compared by Wilcoxon signed-rank test and Student’s *t* test. A difference at *p* < 0.05 was considered statistically significant.

## Results

### Identification of prognosis-specific necroptosis-related lncRNAs in soft tissue sarcomas

**Fig. 1:**
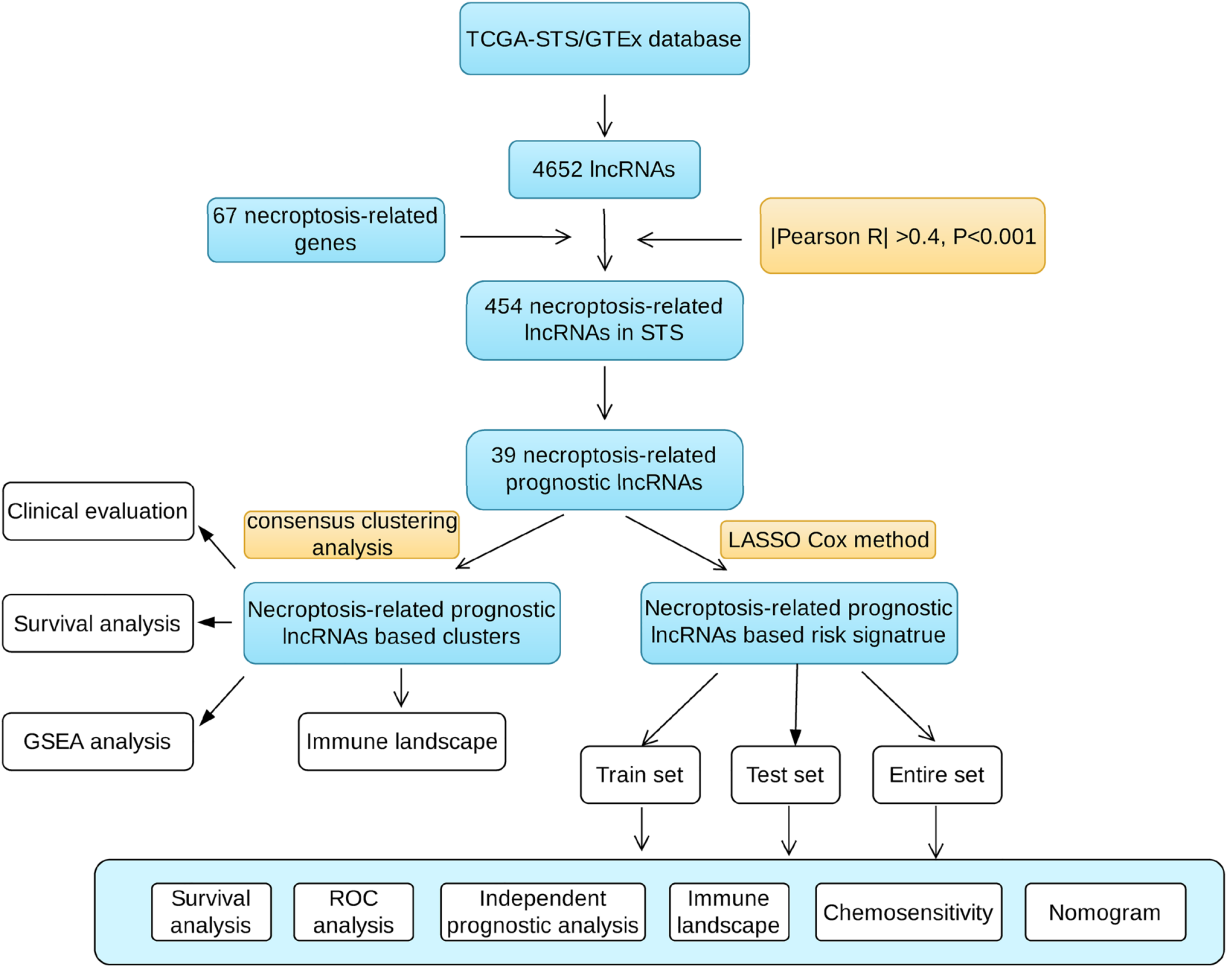
Flow diagram of this study

The general work flow of our study is depicted in Fig. 1. A total of 911 normal soft tissue samples (all from GTEx) and 259 STS patients (all from TCGA) were enrolled in this study. By combining the data from GTEx and TCGA, we collected 4652 lncRNAs. The expression data patterns of 67 NRGs from the GSEA set and relevant literature were acquired, and 454 necroptosis-related lncRNAs was finally extracted using Pearson correlation analysis (|coefficient| > 0.4 and *p* < 0.001). The correlation between these necroptosis-related lncRNAs and corresponding NRGs observed in networks suggests they have an intricate relationship (Fig. S1A). Furthermore, we identified 39 prognosis-specific necroptosis-related lncRNAs with STS via univariate Cox regression analysis (*p* < 0.02) and presented in the forest plot (Fig. S1B). Among these, the expressions of 22 lncRNAs were significantly higher, whereas 17 lncRNAs were lower in tumors (*p* < 0.001; Fig. S1C). Taken together, our study indicated that these prognostic necroptosis-related lncRNAs might play a pivotal role in the progression of STS.

### Unsupervised consensus clustering analysis

**Fig. 2:**
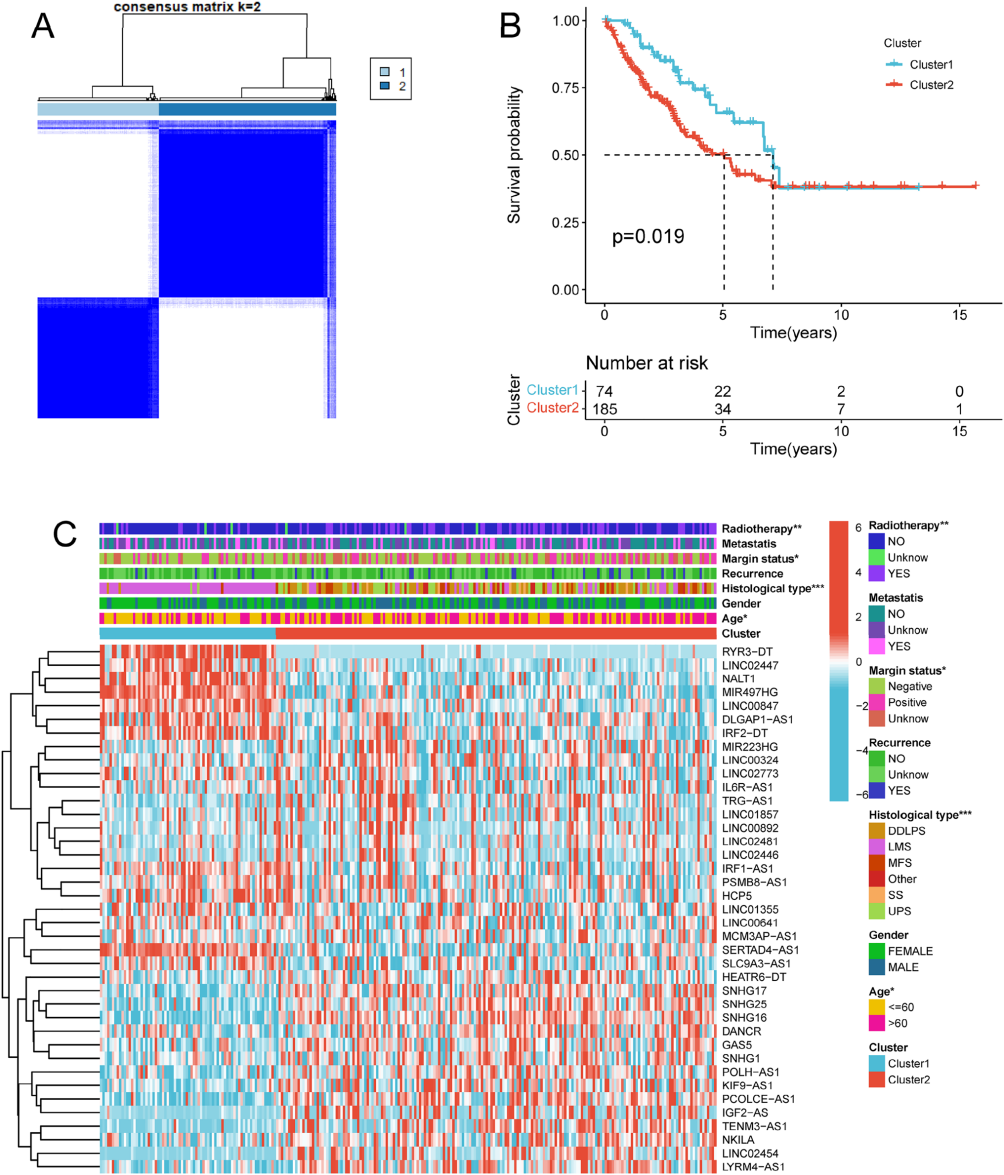
Consensus clustering based on necroptosis-related lncRNAs in STS. **A** Consensus clustering matrix for *k* = 2. **B** Kaplan–Meier analysis of patients in cluster 1 and cluster 2 subgroups. **C** Heatmap and clinicopathologic features of the two clusters (* *p* < 0.05; ** *p* < 0.01; *** *p* < 0.001)

**Fig. 3:**
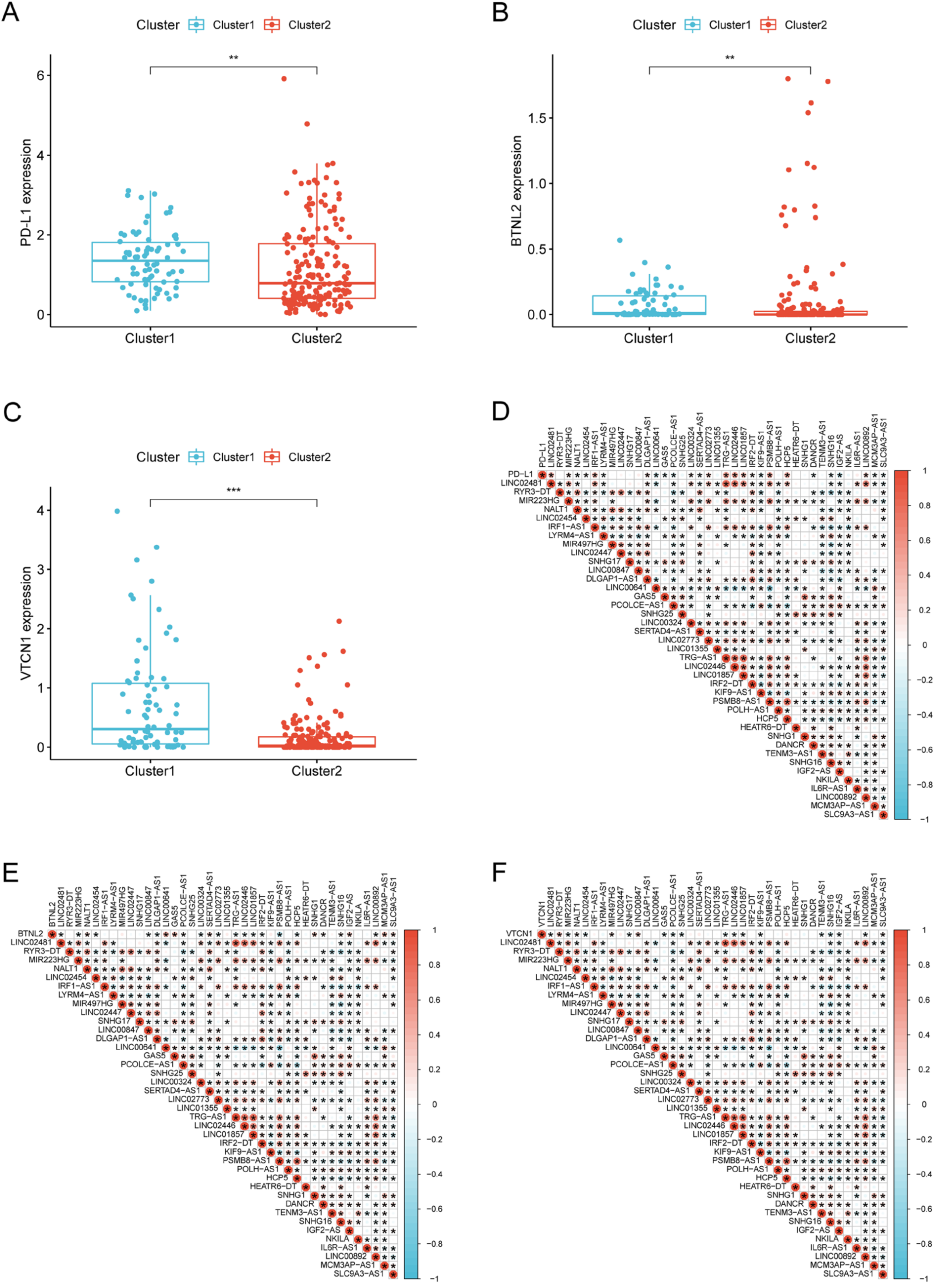
Necroptosis-related lncRNAs were correlated with immune checkpoints in STS. The expression of immune checkpoints **A** PD-L1, **B** BTNL2 and **C** VTCN1 in cluster 1 and cluster 2 subgroups. **D**–**F** Co-expression analysis of immune checkpoints and 39 necroptosis-related lncRNAs

Subclusters in STS identified by unsupervised consensus clustering were reported to have different TME and immunotherapy response (Das et al. 2020; DeBerardinis 2020). *K* = 2 was confirmed as the optimal number of clusters (Fig. 2A). Therefore, hierarchical unsupervised clustering of the 39 necroptosis-related lncRNAs revealed two major clusters (i.e., cluster 1 and cluster 2). Survival analysis evidenced that STS patients in the cluster 2 had a worse OS than those in cluster 1 (Fig. 2B), and a significant differences in age, histological type, margin status and radiotherapy were observed in the heatmap between cluster 1 and cluster 2, indicating the potential correlations between clinical characteristics and 39 necroptosis-related lncRNAs (Fig. 2C). The abundance of eight immune cells, including resting T cells CD4 memory, activated T cells CD4 memory, M0 macrophages, M2 macrophages, resting Dendritic cells, resting Mast cells, Eosinophils and Neutrophils, were significant difference in cluster 1 and cluster 2 (Fig. S2A). The abundance of activated T cells CD4 memory, M0 macrophages, M2 macrophages, Eosinophils and Neutrophils was significantly higher, whereas that of resting T cells CD4 memory, resting Dendritic cells and resting Mast cells was lower in cluster 2 than in cluster 1 (Fig. S2B). This result indicated the potential connection between immune infiltrate cells and 39 necroptosis-related lncRNAs. Meanwhile, the expression levels of several immune checkpoints, including, PD-L1, BTNL2 and VTCN1, were markedly higher in cluster 2 than in cluster 1, whereas that of PD-1 and CTLA4 was lower in cluster 2 than in cluster 1 (Fig. 3A–C; Fig. S3). The correlations between these immune checkpoints and 39 necroptosis-related lncRNA were displayed in Fig. 3D–F. Summing up our observations, the finding of our data showed a significant correlation between necroptosis-related lncRNAs and the prognosis as well tumor-immune landscape in STS.

### Gene set enrichment analysis in subclusters

We further performed GSEA analysis between the subclusters to investigate potential functions of 39 necroptosis-related lncRNAs. The finding of our data illustrated that multiple signaling pathways, including KEGG_APOPTOSIS, KEGG_N_GLYCAN_BIOSYNTHESIS, KEGG_PANCREATIC_CANCER, KEGG_WNT_SIGNALING_PATHWAY, and KEGG_NOD_LIKE_RECEPTOR_SIGNALING_PATHWAY, were remarkably enriched in cluster2, and KEGG_VASCULAR_SMOOTH_MUSCLE_CONTRACTION, KEGG_PROPANOATE_METABOLISM, KEGG_CALCIUM_SIGNALING_PATHWAY, KEGG_INSULIN_SIGNALING_PATHWAY and KEGG_FATTY_ACID_METABOLISM were more enriched in cluster 1 (Fig. S4). These results provided important insights into the potential cross-signaling pathways in STS, which modulated by necroptosis-related lncRNAs.

### Construction and verification of the risk signature

**Fig. 4:**
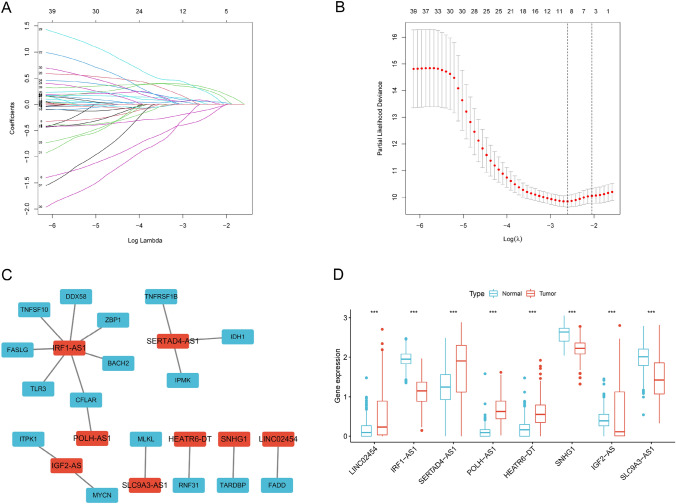
Construction of NRLncSig in STS. **A**,**B** LASSO analysis with minimal lambda value. **C** The relationship between the hub prognostic necroptosis-related lncRNAs and corresponding NRGs. Blue: NRGs. Red: lncRNAs. **D** The boxplot of the hub prognostic necroptosis-related lncRNAs in both tumor and normal samples. NRGs, necroptosis-related genes; NRLncSig, necroptosis-related lncRNA-based risk signature (* *p* < 0.05; ** *p* < 0.01; *** *p* < 0.001)

We finally identified the eight-hub necroptosis-related lncRNAs based on the result of Lasso Cox regression analysis to prevent overfitting (Fig. 4A, B). The relationship between these eight lncRNAs and corresponding NRGs is shown in Fig. 4C. The comparison in the expression of these eight lncRNAs expression suggested that the expressions of LINC02454, SERTAD4-AS1, POLH-AS1 and HEATR6-DT were notably higher, whereas IRF1-AS1, SNHG1, IGF2-AS and SLC9A3-AS1 were more lowly expressed in tumor samples in comparison with normal soft tissue samples (*p* < 0.001; Fig. 4D). Furthermore, the risk score of every STS patient is calculated in the formula as follows:

$${\text{Risk}}\,{\text{score}} = {\text{LINC}}02454\,{\text{exp}}. \times (0.0491) + {\text{IRF}}1 - {\text{AS}}1\,{\text{exp}}. \times ( - 0.3121) + {\text{SERTAD}}4 - {\text{AS}}1\,{\text{exp}}. \times ( - 0.1332) + {\text{POLH}} - {\text{AS}}1\,{\text{exp}}. \times (0.3079) + {\text{HEATR}}6 - {\text{DT}}\,{\text{exp}}. \times (0.2914) + {\text{SNHG}}1\,{\text{exp}}. \times (0.0858) + {\text{IGF}}2 - {\text{AS}}\,{\text{exp}}. \times (0.0572) + {\text{SLC}}9\,{\text{A}}3 - {\text{AS}}1\,{\text{exp}}. \times (0.3491)$$

**Fig. 5:**
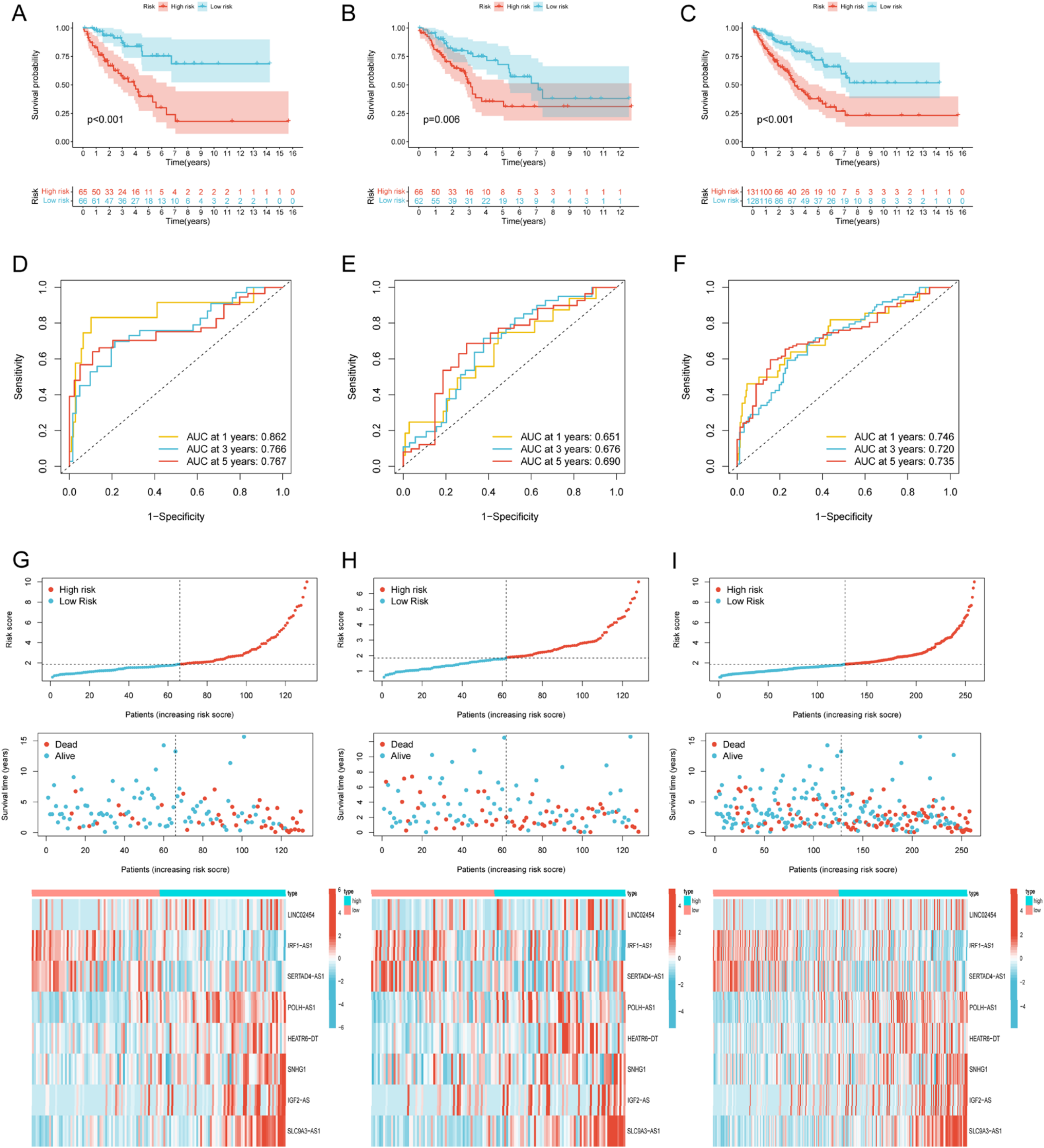
Prognosis value of the NRLncSig in the train, test, and entire sets. **A**–**C** Kaplan–Meier survival curves of overall survival of patients between low- and high-risk groups in the train, test, and entire sets, respectively. **D**–**F** The 1-, 3-, and 5-year ROC curves of the train, test, and entire sets, respectively. **G**–**I** Survival time, survival status and the hub lncRNAs expression between low- and high-risk groups in the train, test, and entire sets, respectively. NRLncSig, necroptosis-related lncRNA-based risk signature

We defined this risk signature as NRLncSig. The prognostic performances of the NRLncSig were evaluated using external validation, including the test set (*n* = 128) and entire set (*n* = 259). The STS patients could be clearly divided into low- and high-risk groups in each set based on the medium risk score. The survival analysis suggested that high-risk group had a worse prognosis whether in the train, test, or entire sets (Fig. 5A–C). Time-dependent receiver operating characteristics (ROC) curve illustrated that the area under the ROC curve (AUC) of 5-year AUC in the train set, test set and entire set was 0.767, 0.690, and 0.735, respectively (Fig. 5D–F). Besides, with the risk score formula, the distribution of risk score, the survival status, survival rate, and the corresponding expression data of these hub lncRNAs of patients were compared between low- and high-risk groups in the train, test, and entire sets (Fig. 5G–I). The results all evidenced that the high-risk group had the worse OS.

### Clinical evaluation of the NRLncSig

**Fig. 6:**
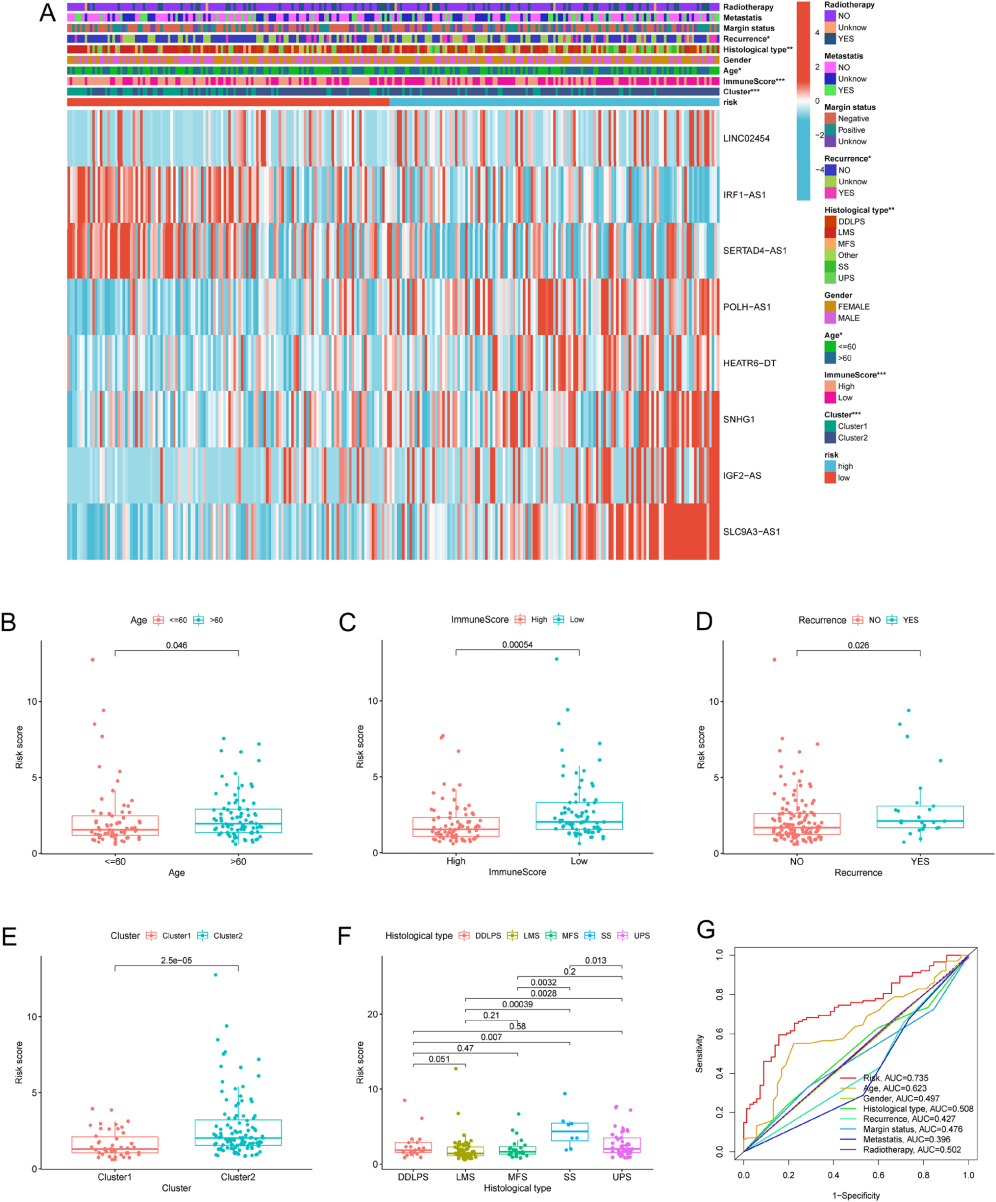
Clinical evaluation of NRLncSig in STS. **A** The heatmap of the associations between the NRLncSig risk score and clinicopathological features. The boxplots of the associations between the risk score and **B** age, **C** immune score, **D** confirmed recurrence, **E** cluster and **F** histological type. **G** A comparison of 5-year ROC curve with other clinical characteristics. NRLncSig, necroptosis-related lncRNA-based risk signature (* *p* < 0.05; ** *p* < 0.01; *** *p* < 0.001)

We performed survival analyses on the NRLncSig of patients with different age, sex, recurrence, metastasis, radiation therapy, margin status, and histological type to further verify the prognostic value of the NRLncSig in various STS patients. High-risk group was confirmed to have a worse OS for both male and female patients, regardless of whether they were ≤60 or >60 years old, with or without metastasis, with positive margin status or negative margin status, with or without recurrence, and whether they were patients with DDLPS and LMS (Fig. S5A–M). These results above again verified the promising prognostic predictor of the NRLncSig for STS patients, regardless of other clinical features. The relationship between the NRLncSig and clinical features was also investigated and the results showed that the NRLncSig score was observed to be significantly connected with recurrence, histological type, age, immune score, and clusters (Fig. 6A). The risk score of patients in age > 60 presented higher risk scores than those in age ≤ 60; patients with high immune scores presented a lower risk score than those with low immune scores. Patients with recurrence were higher than that of patients without recurrence; patients in cluster 2, who exhibited poor OS, presented higher risk scores than those in cluster 1; and patients with SS had a remarkably higher risk scores than those with DDLPS, LMS, MFS and UPS (Fig. 6B–F). In addition, the NRLncSig had a higher AUC of 5-year comparing with other clinical features (AUC = 0.735; Fig. 6G). All the results above illustrated that the accuracy of NRLncSig was a satisfactory and reliable prognostic predictor.

### Gene set enrichment analysis based on risk subgroups

**Fig. 7:**
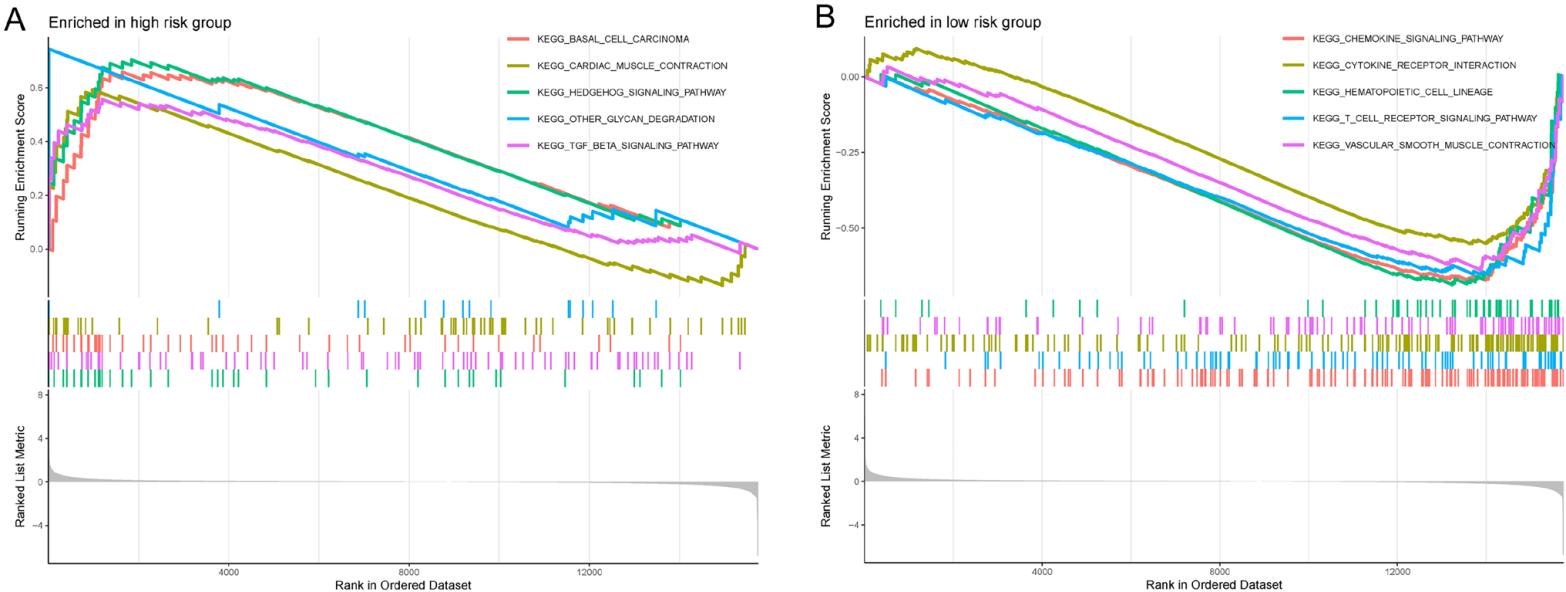
Gene set enrichment analysis in the high- and low-risk groups. Top five enriched pathways enriched for high- (**A**) and low-risk scores (**B**), respectively

The results of GSEA indicated that the KEGG_BASAL_CELL_CARCINOMA, KEGG_CARDIAC_MUSCLE_CONTRACTION,KEGG_HEDGEHOG_SIGNALING_PATHWAY, KEGG_OTHER_GLYCAN_DEGRADATION, and KEGG_TGF_BETA_SIGNALING_PATHWAY were the top five most enriched signaling pathways in the high-risk group, while the KEGG_CHEMOKINE_SIGNALING_PATHWAY, KEGG_CYTOKINE_RECEPTOR_INTERACTION, KEGG_HEMATOPOIETIC_CELL_LINEAGE, KEGG_T_CELL_RECEPTOR_SIGNALING_PATHWAY and KEGG_VASCULAR_SMOOTH_MUSCLE_CONTRACTION were most enriched in the low-risk group (Fig. 7A, B; Table S2). These results also provided novel insights into the potential pathways in STS, which was regulated by eight-hub necroptosis-related lncRNAs.

### The immune landscape based on risk subgroups

**Fig. 8:**
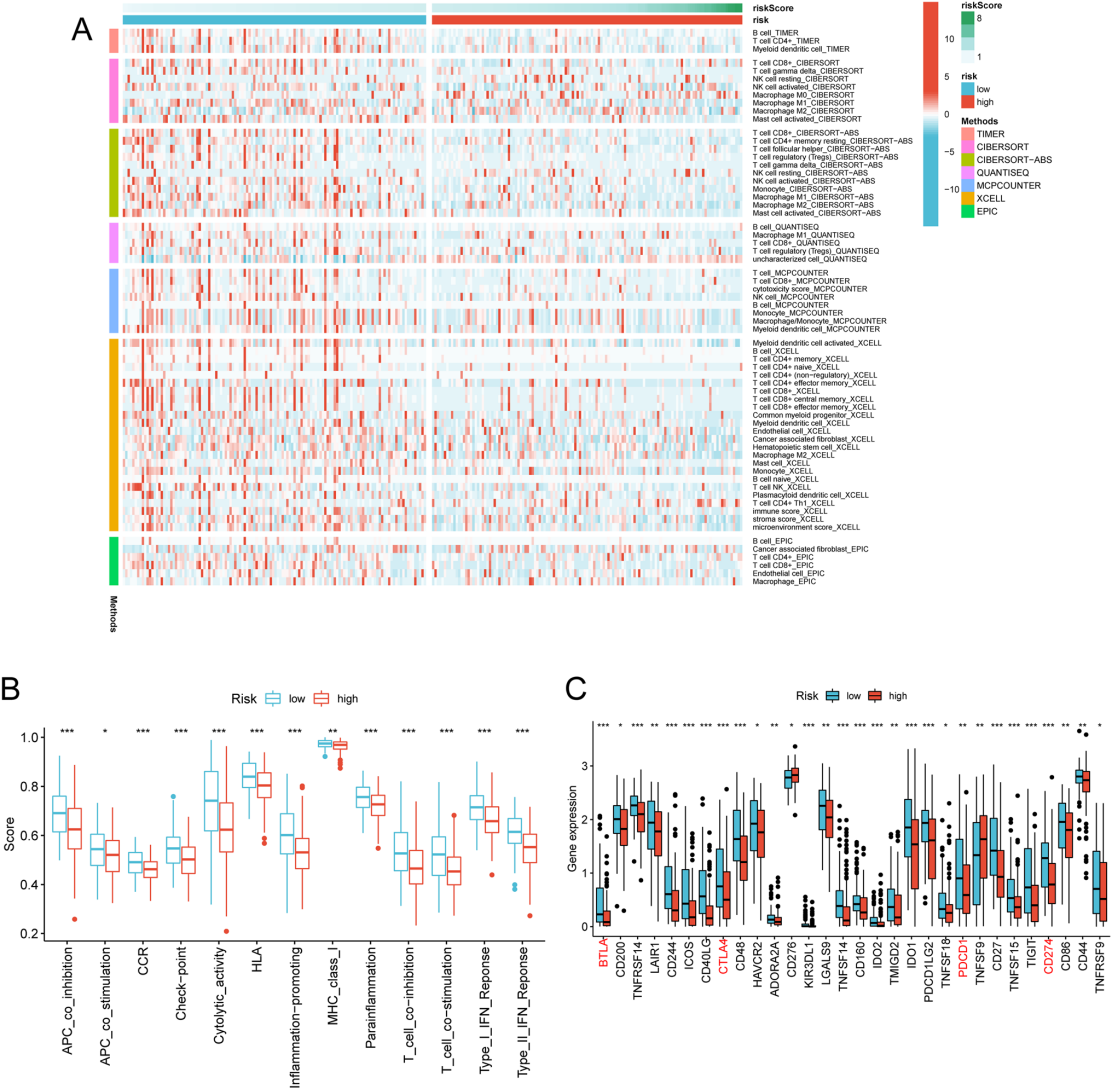
The NRLncSig was correlated with immune cell infiltrates in STS. **A** Heatmap for immune responses based on CIBERSORT, CIBERSORT-ABS, MCP counter, QUANTISEQ, XCELL, EPIC and TIMER algorithms among high- and low-risk groups. **B** ssGSEA for the association between immune cell subpopulations and related functions. **C** Expression of immune checkpoints including PDCD-1 (PD-1), CD274 (PD-L1) CTLA4 and BTLA, among high- and low-risk groups. NRLncSig, necroptosis-related lncRNA-based risk signature (* *p* < 0.05; ** *p* < 0.01; *** *p* < 0.001)

The heatmap of immune cell infiltrates according to diverse algorithms, including CIBERSORT, CIBERSORT-ABS, MCP counter, QUANTISEQ, XCELL, EPIC and TIMER, is demonstrated in Fig. 8A. Most significant immune cells were correlated with the low-risk group on diverse algorithms displayed at the heatmap, including macrophage M2, T cell CD4+ naive, immune score at XCELL, B cell, T cell CD4+ at TIMER, T cell CD8+ at QUANTISEQ and macrophage at MCPcounter and EPIC (Table S3). As we know, the extent of immune cell infiltration between ‘hot’ and ‘cold’ tumors differs substantially. All the results indicated the low-risk group had a higher immune infiltration status and behaved as the ‘hot’ tumor. The ssGSEA analysis showed that all the 13 immune functions were notably different between low- and high-risk groups, and the T cell functions including checkpoint, cytolytic, HLA, regulation of inflammation, co-stimulation of T cell, co-inhibition of T cell, type II INF response and type II INF response were significantly higher in the low-risk than of high-risk groups (Fig. 8B).

Checkpoint inhibitors are the most thoroughly investigated class of immunotherapy to date and have transformed clinical management for a number of malignancies, such as STS. Given that the importance of cancer immunotherapy with immune checkpoint inhibitors, we further investigated the difference in the expression of immune checkpoints between the high-risk and low-risk groups and the result revealed that all 30 immune checkpoints were more active in the low-risk group, including PDCD-1 (PD-1), CD274 (PD-L1), CTLA4, LAG3, and BTLA (Fig. 8C). A negative correlation was further observed between the TME score and NRLncSig score (Fig. S6A–C) and the results indicated that the low-risk group had a higher immune score, stromal score and ESTIMATE score, which signifying a different TME from the high-risk group. Moreover, the NRLncSig score was positively with M2 macrophages, M0 macrophages, Neutrophils, memory B cells and resting NK cells, while negatively associated with M1 macrophages, resting Dendritic cells, regulatory T cells (Tregs), CD8+ T cells, resting Mast cells, naïve B cells and gamma delta T cells (Fig. S6D). These finding above evidenced that the high-risk group presented the immunosuppressive microenvironment in STS, prompting further prognosis research as well providing clinical treatment guidance.

### The assessment of immunotherapy response and chemosensitivity in risk subgroups

**Fig. 9:**
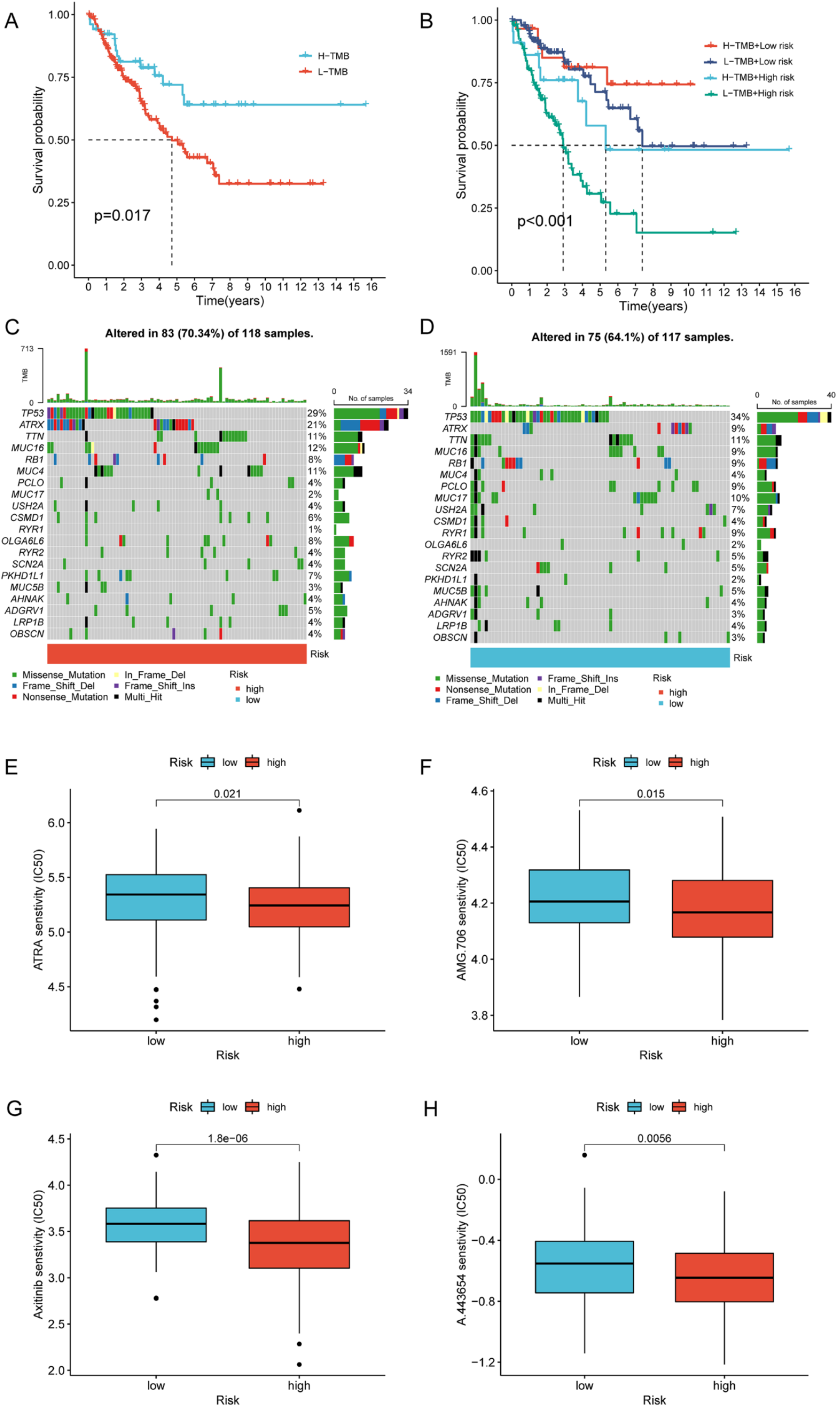
The NRLncSig was correlated with tumor mutation burden (TMB) and chemosensitivity in STS. **A** The survival between high- and low-TMB groups in the entire set was analyzed using Kaplan–Meier curves. **B** The combination of TMB and NRLncSig risk score helped predict the overall survival. **C**,**D** The waterfall diagram was constructed based on different NRLncSig risk groups, blue for low and red for high. **E**–**H** The correlations between the NRLncSig and chemosensitivity in STS. NRLncSig, necroptosis-related lncRNA-based risk signature

We further investigated the potential of NRLncSig score as predictor for immunotherapy response. We divided 259 STS patients into the high- and low-TMB group based on the optimal cutoff and evaluated the prognosis in these subgroups, which revealed that low-TMB patients had a worse OS comparing with the high-TMB patients (Fig. 9A). The combination of high-TMB and low-risk score in NRLncSig had the best OS in STS via survival analysis (Fig. 9B). This prompted us to postulate a correlation of necroptosis-related lncRNA and TMB and they cumulatively contribute to prognosis of STS patients. The waterfall diagram showed that most of the mutated genes in STS patients were higher in the high NRLncSig score group (70.34%) compared with the low score group (64.10%) (Fig. 9C, D). The top five genes with the highest mutation frequencies were TP53, ATRX, TTN, MUC16 and RB1. To investigate whether the NRLncSig possess the potential to predict chemosensitivity of patients with STS, we evaluated its chemotherapeutic efficacy. We found that higher IC50 value of some chemotherapeutic agents in low-risk patients, such as Motesanib, Axitinib and A.443654, indicating that patients with low risk were more sensitive to these chemotherapeutic drugs (Fig. 9E–H). The results suggested that the low-risk group characterized by a higher immune score, had a higher IC50 of aforementioned drugs. Results above suggested the promising potential of NRLncSig to predict response immunotherapy and chemotherapy efficacy of STS patients.

### Construction and verification of the nomogram

**Fig. 10:**
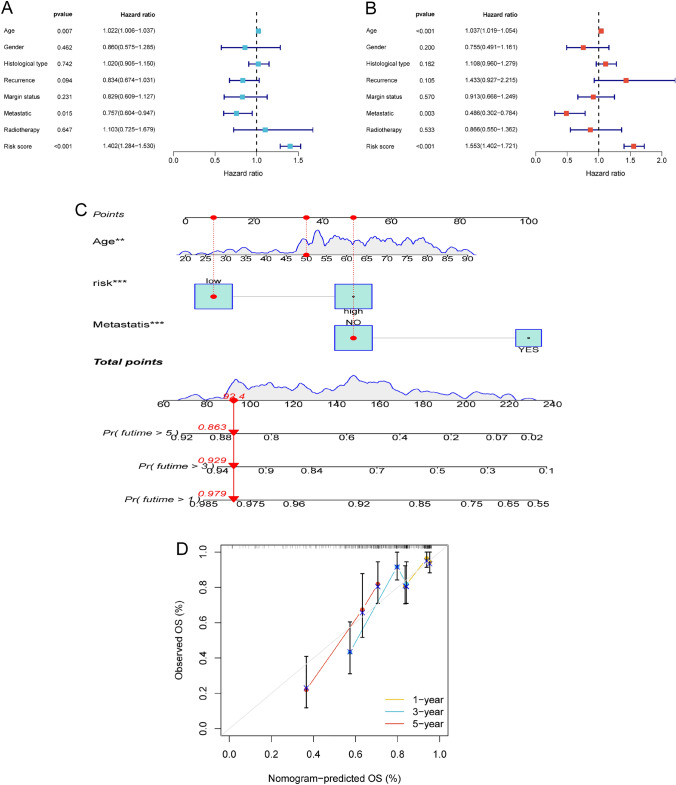
A prognostic signature-based nomogram for predicting 1-, 3-, and 5-year survival in STS patients. **A**,**B** Univariate and multivariate Cox analyses of the NRLncSig risk score and clinical variables in the entire set. **C** A nomogram for predicting survival in the entire set. **D** Nomogram calibration plots for predicting overall survival at 1, 3, and 5 years in the entire set

The hazard ratio (HR) of the NRLncSig and 95% confidence interval (CI) were 1.402 and 1.284–1.530 (*p* < 0.001), respectively, in univariate Cox regression while 1.553 and 1.402–1.721 (*p* < 0.001), respectively, in multivariate Cox regression (Fig. 10A, B). Besides, age and metastasis were observed in the forest plot as independent prognostic predictors. We established the novel nomogram combined with age and metastasis for predicting the 1-, 3-, and 5-year survival of STS patients (Fig. 10C). The calibration curve of 1-, 3-, and 5-year demonstrate that the nomogram had a good accuracy for predicting OS of individual STS patients (Fig. 10D). For this reason, this nomogram is a good predictive tool and might be have the potential for clinical applications in future.

## Discussion

In recent years, molecular markers have shown great potential in tumor screening, diagnosis, efficacy prediction, disease course monitoring, recurrence, metastasis and prognosis. Tumor biomarkers include biological functional molecules such as proteins and nucleic acids, which can specifically reflect the occurrence and development process of tumors. Its accurate and highly sensitive quantitative detection have promising potential for clinical diagnosis, therapy selection decision and prognosis evaluation. With the further exploration on the mechanism of lncRNA in tumorigenesis and development, the diagnostic efficacy of lncRNA in the progression and prognosis of STS is improving. In recent years, many reports have shown that abnormal expression of lncRNA has emerged as critical regulators even oncogenic or tumor suppressor genes in the cancer of various types (Bhan et al. 2017; Qi and Du 2013; Liang et al. 2020; Loewen et al. 2014; Lorenzi et al. 2019; Schmitz et al. 2016). Due to the existence of innate antiapoptotic activities of tumors, we have to consider improve clinical strategy by inducing other anti-tumor effects mechanisms including necroptosis to improve clinical strategy (Tang et al. 2020). Recently, a study have constructed a prognosis signature based on necroptosis-related lncRNAs for gastric cancer (Zhao et al. 2021). Integrating necroptosis and lncRNAs, Zhao et al. tried to establish a clinical model which discriminating the cold and hot tumors by tumor heterogeneity and the different immunologic landscape for further risk-stratification to evaluate prognosis and improve clinical decision. Therefore, focusing on the biological significance of necroptosis and lncRNAs in tumors may help fill in the knowledge gap and open new scenario in the field of immunotherapy (Zhao et al. 2021). However, few studies have explored abnormalities in genomic and tumor immunology based on a perspective integrating necroptosis-related lncRNAs and no studies have established risk signature associated with necroptosis-related lncRNAs in STS. Therefore, the exploration of necroptosis-related lncRNAs for STS presents a broad prospect of clinical application. As far as we know, this study is the first of its kind to focus on necroptosis-related lncRNAs and try to establish signature based on rigorous theory for clinical application in STS.

In this paper, we were the first time to investigative the influence of necroptosis-related lncRNAs in STS. A total of 259 STS patients were included in the study. We systematically selected 454 lncRNAs and further investigated their interactions and established correlation network, after that, 39 candidate necroptosis-related lncRNAs with important prognostic value were selected from them. We next performed clustering methods and divided those STS patients into 2 clusters and noticed significantly distinct in immune landscape and clinical outcome in the two clusters. Worse prognoses were noted in cluster 2, suggesting relevance of necroptosis-related lncRNAs to prognosis, and possess potential as an independent prognosis biomarker in STS. Another important result was that cluster 2 exhibited higher expression of PD-L1, BTNL2 and VTCN1, while lower expression of PD-1 and CTLA4 than cluster 1. The expression of necroptosis-related lncRNAs including LINC02454, IGF2-AS, and SLC9A3-AS1 had significant correlation with immune checkpoint expression, showing a potential regulatory role of necroptosis-related lncRNAs in immune checkpoints. LncRNAs can affect tumor immunity by influencing the type, distribution, number and functional status of immune cells. It is documented in the literature that lncRNAs play a regulatory role in many aspects of tumor immunity (Huang et al. 2018). Hence, we conducted further study to the immune infiltration cells in subclusters. The abundance of activated T cells CD4 memory, M0 macrophages, M2 macrophages, Eosinophils and Neutrophils was significantly higher, whereas that of resting T cells CD4 memory, resting Dendritic cells and resting Mast cells was lower in cluster 2 than in cluster 1 in this research. Some studies have demonstrated that CD4+ T cells in infiltrating lymphocytes occupying core proportions in the microenvironment were related to prognosis in sarcomas. Alspach and Ostroumov et al. have reported that CD4+ T cells lymphocyte activation is essential to immune checkpoint inhibitors (Ostroumov et al. 2018; Alspach et al. 2019). The result indicated that the immune landscape was different in subcluster in STS. The GSEA results further revealed that necroptosis-related lncRNAs appear to serve key roles in immune-related processes in the course of tumor progression. Those exciting discoveries prompted us to further explore the roles of necroptosis-related lncRNAs in tumor immunity.

We established the NRLncSig score and performed survival analysis, performance evaluation as well a series of correlation analyses to explore the possible roles of necroptosis-related lncRNAs in STS, and the result showed that patients in the high-risk group had a worse prognosis. The subgroup analyses also confirmed NRLncSig with independent prognostic significance in STS. The 5-year of AUCs in the train, test and entire set were 0.767, 0.690, 0.735, respectively, suggesting that robust predictive abilities of NRLncSig for STS patients.

The eight necroptosis-related lncRNAs of the NRLncSig including LINC02454, SERTAD4-AS1, POLH-AS1, HEATR6-DT, IRF1-AS1, SNHG1, IGF2-AS and SLC9A3-AS1 selected by a series of stepwise screening assays. Most of them had been shown to be related with the OS in STS (Fig. S7) and implicated in tumor occurrence and development. For example, Tan et al. reported the notably high expression and possible contribution as an oncogene of LINC02454 in thyroid cancer by the exploration of microarray profiling and in-vitro studies. SNHG1 has been reported to up-regulated MTDH by sponging miR-145-5p and led to non-small cell lung cancer progression (Lu et al. 2018). Okutsu reported the aberrant expression of IGF2-AS in Wilms’ tumor and suggested the potential of IGF2-AS as one of the candidate Wilms’ tumor genes (Okutsu et al. 2000), while research about SLC9A3-AS1 reported the carcinogenic effect on NPC cells exerted by SLC9A3-AS1 through adjusting the miR-486-5p/E2F6 axis (Li et al. 2021).

TME has recently emerged as a critical part in development and tumorigenesis. In our study, patients with high immune scores had lower NRLncSig risk scores than those with low immune scores and negatively related to the risk score. Previous researches indicated that a high immune score generated by ESTIMATE algorithm represents a favorable outcome, which was consistent with our results (Hu et al. 2020; Wang et al. 2021). In addition, results of the analyses of immune microenvironment of STS revealed that the NRLncSig risk score was positive with M2 macrophages, M0 macrophages, Neutrophils, memory B cells and resting NK cells, while negatively related to M1 macrophages, resting dendritic cells, regulatory T cells (Tregs), CD8+ T cells, resting Mast cells, naïve B cells and gamma delta T cells. Previous findings have shown that infiltrating immune cells in the tumor affected prognoses of patients. For instance, Tregs closely correlated with anti-cancer immune responses and the cancer immunity cycle, and higher percentages of Tregs lead to better survival in a number of tumor types, covering STS (Peng and Menaka 2021; Rocha et al. 2021). Previous studies have revealed that a higher abundance of macrophage M0 was significantly correlated with worse prognosis and clinical outcomes, whereas an abundance of macrophage M1 led to a reliable prognosis in STS (Fan et al. 2021). Those findings of other studies are in line with the results from this study and suggest that necroptosis-related lncRNAs play their important regulatory role by influencing the type, distribution, number and functional status of immune cells. The ssGSEA analysis revealed that all the 13 immune functions were notably different between low- and high-risk groups, and the low-risk group stand obviously more active T cell-related functions than high-risk one. This suggests that the low-risk group is in an anti-tumor state through immune activation, and necroptosis-related lncRNAs may be the crucial regulation cores of immune cell infiltration in STS.

ICBs therapy had shown their promising anti-tumor effect, and it failed to elicit better clinical outcomes for patients with STS, which could be due to unclear expression pattern and effect of immune checkpoint in STS (Filipa et al. 2021). Therefore, we further evaluated the correlations between the NRLncSig risk score and immune checkpoint expression. Results showed a significant correlations of risk score and expression of some immune checkpoints including PDCD-1 (PD-1), CD274 (PD-L1), CTLA4, LAG3, BTLA and interestingly, the substantial upregulation of above immune checkpoints had been observed in the low-risk group. PD-L1 is an immunoinhibitory molecule that suppresses the activation of T cells, leading to the progression of tumors (Wang et al. 2016). The physiological function of PD-L1 is to bind to the PD-1 receptor on T cells and transmit immunosuppressive signals so as to inhibit the activity of effector T cells, preventing them from killing tumor cells (Zhou et al. 2020). In addition, Tregs has also been reported to be affected by PD-1/PD-L1 blockade (Cai et al. 2019). Our risk signature showed its potential as predictive biomarker for immune checkpoint expression and response to immune checkpoint blockade (ICBs) in STS.

Several recent studies have shown that tumors with high TMB tend to have high CD8+ immune cell infiltration levels, forming a favorable immune environment. It is widely accepted and become a consensus that TMB can serve as the predicting factors for the efficacy of ICBs (Rizvi et al. 2015; McGranahan et al. 2016). Besides, high TMB was reported to activate host anti-cancer immune responses and be associated with favorable prognosis (Cristescu et al. 2018; Seiwert et al. 2016). Taking value of TMB in immunity and immunotherapy into consideration, we analyzed somatic mutation data of samples in TCGA-SARC cohort, exploring the correlation of STS-TMB and risk score. And we found that OS of patients with high TMB was relatively favorable. Followed stratified survival analysis showed that combination to TMB did not influence independence of risk score as prognosis factors suggested an excellent ability to risk- stratification of TMB-risk score joint model. Results also suggested worst OS of patients with low-TMB and high-risk score. Furthermore, results also show that TMB rate of high-risk score group was significantly higher than in low-risk one. Our observations and discoveries might offer a novel perspective for immune checkpoint blockade therapy.

Chemotherapy is a widely used strategy in cancer treatment with unsatisfactory efficacy due to chemoresistance, thus reducing the chemoresistance issues and expanding the field of immunotherapy are also of primary importance except identify effective therapeutic target. Our work that observed differences in sensitivity to various chemotherapeutic agents including ATRA, Motesanib, Axitinib and A.443654 in subgroups with high and low scores may open up a possibility for that. NRLncSig may be applied to predict chemosensitivity and evaluate the response of STS patients to chemotherapy, coming to improve clinical strategy. Moreover, these hub necroptosis-related lncRNAs has shown great potential to be candidate targets for improving the effectiveness of chemosensitivity.

There is urgent need for a clinical prognosis tool that is not only reliable and accurate but also practical and intuitive and it gives rise to nomogram, a mathematical scoring system (Balachandran et al. 2015). By consider together several independent prognostic factors, nomogram works out robust predicting results of clinical outcome, including death or disease recurrence (Iasonos et al. 2008). To the best of our knowledge, a NRLncSig-based prognostic tool have not previously been reported. Therefore, we conducted a nomogram that could be used as a novel tool to quantify the prognosis of STS patients by combining NRLncSig risk score with other clinical variables including age and metastasis, and can aid in individualized therapy. The calibration curve of 1, 3 and 5 years showed that the nomogram was a good prognostic tool.

However, we also recognize that there are some limitations to our study that cannot be ignored. First, our research was based on public databases, so our access to clinical information and gene expression data is limited; second, our research relies heavily on computational analyses, and additional vivo and vitro experiments are important to verify these results above in the future.

## Conclusions

In summary, we established a novel NRLncSig which showed its advantage in identifying significant distinction in patients with different immune landscape, TMB, therapeutic efficacy of immunotherapy and clinical outcome in STS, providing clinicians a guide to identify and treat high-risk lesions in clinical practice. This research emphasizes critical gaps in knowledge and future prospects for the regulatory roles of necroptosis-related lncRNAs in the tumor immune and also provides new targets and strategies for improving the effectiveness of tumor immunotherapy.

## Supplementary Information

Below is the link to the electronic supplementary material.

Supplementary file1 (DOCX 2096 KB)

## Data Availability

Publicly available datasets were used in this study. These data can be downloaded in the UCSC Xena database (https://xenabrowser.net/).
